# Interfacial-Water-Modulated Photoluminescence of Single-Layer WS_2_ on Mica

**DOI:** 10.3390/ijms24043492

**Published:** 2023-02-09

**Authors:** Yanghee Kim, Haneul Kang, Myeongin Song, Hyuksang Kwon, Sunmin Ryu

**Affiliations:** 1 Department of Chemistry, Pohang University of Science and Technology (POSTECH), Pohang 37673, Republic of Korea; 2 Korea Research Institute of Standards and Science, Daejeon 34113, Republic of Korea

**Keywords:** exciton-exciton annihilation, trion, interfacial water, 2D semiconductor, WS_2_, photoluminescence

## Abstract

Because of their bandgap tunability and strong light–matter interactions, two-dimensional (2D) semiconductors are considered promising candidates for next-generation optoelectronic devices. However, their photophysical properties are greatly affected by their surrounding environment because of their 2D nature. In this work, we report that the photoluminescence (PL) of single-layer WS_2_ is substantially affected by interfacial water that is inevitably present between it and the supporting mica substrates. Using PL spectroscopy and wide-field imaging, we show that the emission signals from A excitons and their negative trions decreased at distinctively different rates with increasing excitation power, which could be attributed to the more efficient annihilation between excitons than between trions. By gas-controlled PL imaging, we also prove that the interfacial water converted the trions into excitons by depleting native negative charges through an oxygen reduction reaction, which rendered the excited WS_2_ more susceptible to nonradiative decay via exciton–exciton annihilation. Understanding the role of nanoscopic water in complex low-dimensional materials will eventually contribute to devising their novel functions and related devices.

## 1. Introduction

Water confined in a reduced dimension exhibits unconventional properties because of the dominant molecular interactions with the confining walls. The liquid–ice phase transition of water in carbon nanotubes is governed by the detailed balance between water–water and water–wall interactions [1,2]. Water flow through carbon nanotubes is a few orders of magnitude faster than what continuum hydrodynamics predicts [3]. The dielectric response of nanometer-thin water sandwiched between inorganic two-dimensional (2D) crystals is severely compromised because of the wall-dictated structural ordering that suppresses molecular rotation [4]. Single-(1L) and few-layer interfacial water sandwiched between graphene and substrates is rigid [5] and yet allows redox reactions that consume the charge carriers of the graphene wall [6,7]. The ubiquitous presence of interfacial water within the assemblies of low-dimensional materials raises more questions beyond those answered already.

One question of priority and yet lacking a clear understanding is how the electronic excitation in low-dimensional materials is affected by the tiny amount of water. This issue has been tackled by a few reports that used interfacial water entrapped between 2D semiconductors and solid substrates. Varghese et al. [8] observed that the photoluminescence (PL) of 1L MoS_2_ on mica is modulated by the presence of interfacial water. The direction of charge transfer responsible for the PL change was dependent on the nature of the substrates and the morphology of the water layers. Park et al. [6] showed that sub-monolayer water accommodates an oxygen reduction reaction (ORR) that injects electrical holes in 1L WS_2_ supported on SiO_2_ and subsequently amplifies the excitonic PL. By exploiting the PL characteristics of 1L WS_2_, Kang et al. [9] observed the diffusion of molecular oxygen through the interfacial water layer in real time. As demonstrated by these studies, a nanoscopic amount of water can have a substantial effect on the decay of excitons in low-dimensional materials. At the same time, it is also natural to expect that there can be other roles of interfacial water that have not been observed. Such exploration can be best performed using a TMD/mica system. Despite their low photoluminescence (PL) quantum yields, the 1L transition metal dichalcogenides (TMDs) are highly useful emitters because of the visible and NIR excitons with large binding energies [10,11]. Mica not only provides an atomically flat surface for 2D crystals [12] but also holds well-ordered water layers because of its crystallinity and large hydrophilicity [13,14].

In this work, we report that interfacial water accelerates the relaxation of high-density excitons in 1L WS_2_ supported on mica substrates. The sublinear power dependence of the PL signals originating from neutral and charged excitons showed that exciton–exciton annihilation (EEA) is an additional quenching channel and more dominating with interfacial water. Wide-field PL imaging revealed that water reduced the net electron density in WS_2_ by an ORR. We conclude that subsequent charge-neutral WS_2_ suffers more from EEA than charged WS_2_ from trion-trion annihilation (TTA). The findings of the current study will benefit fundamental research on excitonic dynamics in heterostructured systems and devising applications based on the control of excitonic fates.

## 2. Results and Discussion

**Figure 1:**
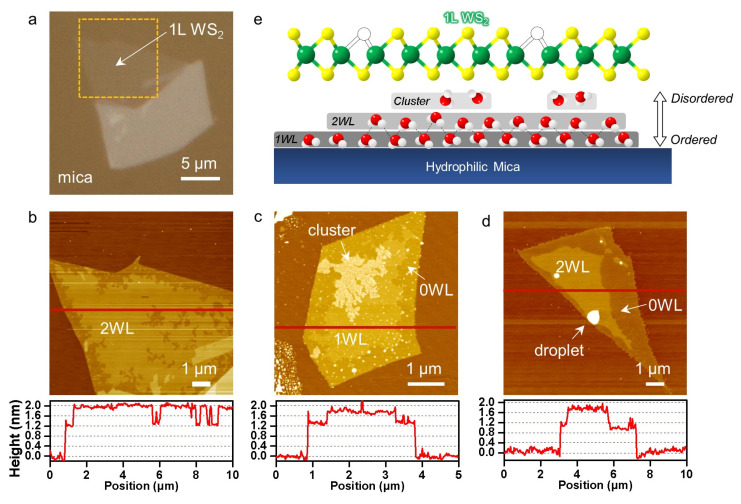
Morphology of the interfacial water layers. (**a**,**b**) Optical micrograph (**a**) and AFM height image and profile (**b**) of 1L WS_2_ on mica. The AFM image showing 2L interfacial water (2WL) was obtained from the orange square in (**a**). The height profile in (**b**) was taken along the red line in (**b**). (**c**,**d**) AFM images and height profiles of two additional samples containing various forms of interfacial water. (**e**) Scheme of 1L WS_2_/water/mica.

Single-layer (1L) WS_2_ samples prepared on mica substrates typically spanned several microns across and produced a significant optical contrast, as shown in Figure 1a. The AFM height images revealed plateau-like structures characteristic of interfacial water layers [13,14]. In Figure 1b, most of the 1L WS_2_ area was found to be ~0.7 nm elevated compared to the water-free areas (0WL) near the edges. Notably, the average elevation was twice the interplanar distance of the hexagonal ice, thus corresponding to water bilayers (2WL) [14]. As confirmed for the graphene/mica system [13,14], the plateaus were attributed to water layers formed during the exfoliation step. The surface of mica is extremely hydrophilic and adsorbs water vapor in the air. As the WS_2_ layers were laminated on top of the water-rich mica surfaces, the water was spread as flat interfacial layers to minimize the mechanical deformation of 1L WS_2_ with a large Young’s modulus [15]. The sample in Figure 1c showed 0 and 1WL regions in addition to clusters with a width of tens of nm. Even larger structures (blisters) were also found, as shown in Figure 1d. Whereas the molecular-level structure of the interfacial layers is far from being understood, it is likely to be close to that of hexagonal ice, as depicted in Figure 1e [13,14,16].

**Figure 2:**
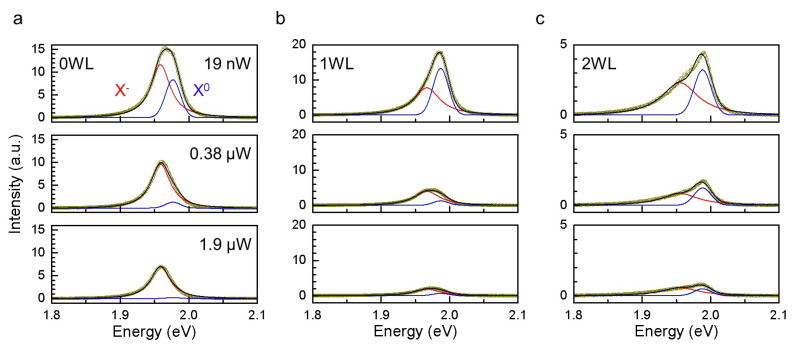
Power-dependent PL spectra of WS_2_/water/mica. (**a**–**c**) A exciton PL spectra (yellow circles) obtained from water-free (**a**), 1WL (**b**) and 2WL (**c**) areas. For each, the average power (P_exc_) of the excitation beam was set to 0.019, 0.38 and 1.9 μW (from top to bottom). Neutral exciton (X^0^) and negative trion (X^−^) were fitted with a Gaussian (blue) and a Lorentzian (red) function, respectively, resulting in an overall fit (black line).

The photoluminescence of 1L WS_2_ is dominated by A exciton (X^0^) and its charged species (X^+^ or X^−^) [6,17]. Because of the prevalent native n-type doping [17], negative trions are mostly observed from exfoliated samples [6,9]. In Figure 2a (top), WS_2_ directly supported on mica also contained the two features that were assembled into an apparently single asymmetric PL band at ~1.96 eV. The dissociation energy of X^−^ turned out to be 18 to 33 meV when fitted with the sum of the Lorentzian and Gaussian functions. The areal ratio between X^−^ and X^0^ (2.79:1) indicated the sample was doped with a significant density of electrons [6]. The polarity of the charges was verified by intentional chemical doping, as will be shown later. When the average power (P_exc_) of the excitation beam was increased (middle and bottom of Figure 2a), the overall PL intensity (I_tot_) decreased notably, specifically, by 55% for a 100-time increase in P_exc_. We noted that even larger decreases were observed in the presence of interfacial water layers, i.e., by ~85% for 1WL (Figure 2b) and by ~80% for 2WL (Figure 2c). As will be discussed below, the reduction in the luminescence yield can be attributed to exciton–exciton annihilation (EEA) [18] that becomes more dominant at a higher density of excitons [19,20,21]. We also noted that the line shape of the PL band changed with increasing P_exc_. The fitted sub-components showed that it was due to the differing power dependence of X^−^ and X^0^, as will be further discussed below.

**Figure 3:**
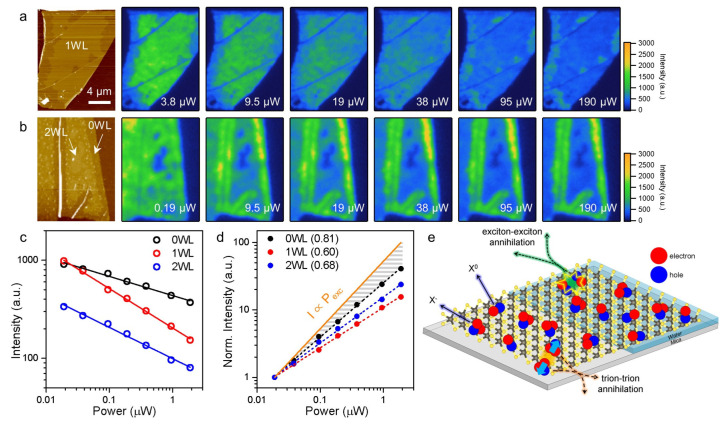
Wide-field PL imaging of power-dependent intensity inversion. (**a**) AFM (leftmost) and PL (the others) images of WS_2_/mica with 0WL and 1WL areas. (**b**) AFM (leftmost) and PL (the others) images of WS_2_/mica with 0WL, 1WL and 2WL areas. Whereas P_exc_ was increased in (**a**,**b**), the corresponding exposure time was decreased reciprocally for a constant photon fluence. (**c**) Integrated PL intensity of the X^−^ and X^0^ peaks for the 0WL, 1WL and 2WL areas of (**b**). Each PL spectrum was obtained for a constant photon fluence while varying P_exc_. Note that P_exc_ for the PL imaging was two orders of magnitude larger than that for the PL spectra in (**c**) because of a large illumination area for the imaging. (**d**) Normalized PL intensity (I) from (**c**) given as a function of P_exc_. Each data point in (**c**) was divided by its exposure time and then normalized with respect to the value at the lowest P_exc_. (**e**) Schematic representation of exciton–exciton annihilation (EEA) and trion–trion annihilation (TTA) in WS_2_/water/mica.

In Figure 3, we adopted wide-field PL microscopy [6] to study the spatial variation of the power dependence of the excitonic emission. Because the two components were spectrally very close to each other, they were collected for imaging without spectral separation. The AFM image in Figure 3a shows that the central 1L WS_2_ region contained 1WL, whereas most of the edge areas were without interfacial water. Then, the PL images in Figure 3a reveal that the 1WL-supported areas produced a stronger PL signal than the water-free region at the lowest P_exc_ of 3.8 μW. With increasing P_exc_ and the simultaneous reduction of the corresponding exposure time, the signals from the 1WL region decreased noticeably. Remarkably, the two regions showed an intensity inversion at the high P_exc_. A similar observation was made for another sample with nWL (n = 0, 1 and 2), as shown in Figure 3b. Compared to the 0WL areas near the edges, 1WL-supported WS_2_ produced progressively less PL intensity with increasing P_exc_.

Figure 3c presents the PL intensity selected from nWL-supported areas (Figure 3b) as a function of P_exc_ after normalization with respect to the fluence of the excitation photons. The log–log intensity plot clearly shows the differing power dependence of the nWL areas. Notably, the 2WL areas also showed a high sensitivity towards P_exc_, which was not readily visible in the PL images (Figure 3b). In Figure 3d, the same data set is given as a function of the photon fluence with each PL signal normalized to the unity at the smallest fluence. The dotted line with a slope of unity represents an imaginary system with a constant PL quantum yield irrespective of P_exc_. Then, the fact that all the series in Figure 3d have smaller slopes indicates that the PL process competed with fast nonradiative decay channels that were affected by the presence of interfacial water. As indicated by the PL images and Figure 3d, single layers of water had the smallest slope of 0.60, which stands in stark contrast to that of the 0WL areas (slope of 0.81).

**Figure 4:**
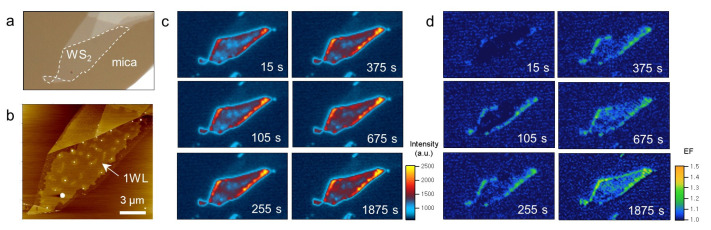
(**a**,**b**) Optical micrograph (**a**) and AFM height image (**b**) of 1L WS_2_/mica. (**c**,**d**) Time-lapse PL images (**c**) and PL enhancement images (**d**) of the sample in (**a**). The sample was pre-equilibrated with Ar gas for 2 h before exposure to Ar/O_2_ mixed gas at time zero. The enhancement factor (EF) was calculated by normalizing each PL image with respect to that at time zero.

Notably, the presence of interfacial water enhanced the nonradiative decay of the excitons in 1L WS_2_. Among the few factors that will be discussed further below, we propose that the observed phenomenon relates to the hole doping of WS_2_, which was induced by the oxygen reduction reaction (ORR), where water plays a key role [6,7]. First of all, we verified that the WS_2_ samples supported on mica (e.g., Figure 4a,b) were also hole-doped, and their excitonic emission was regulated by the ORR as observed for graphene and TMDs supported on hydrophilic silica substrates. Figure 4c,d presents time-lapse PL and its enhancement images of 1L WS_2_ obtained as a function of O_2_ exposure time (t). The enhancement was defined as (I_t_ − I_0_)/I_0_, where I_0_ corresponds to the PL intensity at t = 0. To control the gaseous environment, the samples were mounted in a gas-flow optical cell. Because the cell was purged with high-purity Ar for 2 h before the introduction of O_2_, the PL enhancement can be attributed to the action by O_2_. The earliest image (t = 15 s) in Figure 4d indicates a slight enhancement near some of the edges, and a more prominent change occurred within 2 min. In the following several minutes, we observed a clear edge-to-center propagation of the enhancement fronts. This feature indicated that the PL enhancement was caused by molecular species that diffused through the WS_2_/mica interface from the atmosphere. Considering its reversibility upon exposure to Ar (Figure S1), the reaction behind the overall change was identical to the ORR that was observed for WS_2_ supported on SiO_2_: O_2_ + 4H^+^ + 4e^−^ ⇌ 2H_2_O (acidic) and O_2_ + 2H_2_O + 4e^−^ ⇌ 4OH^−^ (basic) [7]. Whereas the electrons are provided by WS_2_, the water required for the electrochemical reaction can be readily found at the interface formed by hydrophilic substrates such as SiO_2_ and mica in this study. Even the 0WL areas near the edges of Figure 4b included numerous tiny water clusters, possibly with smaller clusters that are not seen in the AFM images.

When natively n-doped WS_2_ is hole-doped by the ORR, the net charge density decreases, and the PL signals will be dominated by neutral excitons rather than trions [6]. Indeed, Figure 2 shows that the contribution of X^0^ was larger in the water-supported areas than in the water-free regions. Then, the nature of EEA readily explains the intensity inversion observed for 1WL-supported and water-free areas. In an EEA process, two excitons collide with each other, and one decays by dumping its energy into the other, which is simultaneously excited further and eventually relaxes nonradiatively [18]. Because of its bimolecularity, EEA becomes more contributing at a higher density of excitons; in other words, P_exc_ is higher. Compared to EEA, TTA is less efficient because of the repulsive interaction between the charged excitons, as depicted in Figure 3e [22]. Indeed, the PL spectra in Figure 2 show that the intensity of X^0^ decreased more than that of X^−^ with increasing P_exc_, irrespective of the presence of interfacial water. Then, the stronger power dependence of the water-supported areas can be attributed to the fact that their PL band was dominated by X^0^, not X^−^.

We noted other factors might be responsible for the observations. First, one may consider that the efficient dielectric screening of water is somehow related. Indeed, energetic relaxation by various solvents has been observed for excitons in TMDs [23]. However, it has recently been shown that the dielectric constant of water is greatly lowered because of water–wall interactions with decreasing dimension [4]. In addition, a few monolayers of water are highly ordered on the surface mica [16,24], which should lead to a further reduction in dielectric screening. Second, interfacial water may induce various degrees of structural corrugation in WS_2_ because 2D materials are prone to out-of-plane deformation and tend to maximize van der Waals interactions with the underlying substrates [25,26]. Such deformed lattice sites may assist the nonradiative decay by acting as centers for momentum exchange required for various scattering processes, as shown in the double-resonant Raman scattering of graphene [27]. We also noted that the morphology of interfacial water may affect the exciton–trion equilibrium. As shown by the samples containing water clusters (Figure 1c), non-flat and three-dimensional forms of interfacial water are more likely to induce a severe deformation of WS_2_ and subsequently larger interfacial voids, which serve as efficient diffusion channels for O_2_ required for the ORR [9]. Compared to hexagonal ice that can form epitaxially on mica, less structured water is also more suitable for the ORR as a solvent because of its enhanced fluidity [16]. Indeed, the PL and enhancement images in Figure 4 show that the WS_2_ areas with high roughness accommodated a more active ORR, which led to a larger exciton/trion ratio and stronger PL signals.

## 3. Materials and Methods

### 3.1. Preparation and Treatments of the Samples

Single-layer WS_2_ samples were prepared at ambient conditions by mechanical exfoliation of bulk WS_2_ crystals (2D Semiconductors, Inc., Scottsdale, AZ, USA) onto freshly cleaved mica substrates (Ted Pella, grade V1 muscovite mica, Redding, CA, USA) [14,28]. Fresh mica surfaces were also prepared by mechanical exfoliation. We identified the thickness of the prepared samples by optical contrast using an optical microscope (Nikon, LV100, Melville, NY, USA) [29]. The relative humidity (RH) was between 19% to 64% at a room temperature of about 22 °C. Some WS_2_ samples were deposited on mica maintained at 40–80 °C using a hot plate. The hot transfer eased the release of the adhesive tape from the substrates and thus facilitated the exfoliation process. However, there was no meaningful correlation between the substrate’s heating and the overall amount of interfacial water.

### 3.2. Topographic Measurements

The AFM (atomic force microscopy) characterizations were performed at ambient conditions in an amplitude-modulated non-contact mode [14]. The probe tips (MicroMasch, NSC-15, Oelsnitz, Germany) were oscillated with a nominal amplitude of 20 nm, and the scan rates were between 0.3 and 0.5 Hz.

### 3.3. Photoluminescence Measurements

PL was obtained by a homebuilt micro-PL spectrometer setup [6] with a grating of 300 grooves/mm at ambient conditions. A solid-state laser operating at a wavelength of 514 nm was used as the excitation source. The laser beam was focused onto a sample (FWHM of the focal spot ~0.60 µm) using a microscope objective (40×, numerical aperture = 0.60). The back-scattered PL signal was collected with the same objective and guided to a spectrometer combined with a charge-coupled device. For wide-field PL imaging, the collimated laser beam was extended 3 times with a Galilean beam expander and then focused on the back-focal plane of the objective with a plano-convex lens (focal length = 500 mm) [6,9]. For homogeneous excitation, the illumination area (FWHM ~45 µm) was maintained one order of magnitude larger than the typical sample. The PL signals in the range between 1.9 and 2.1 eV mostly contributed to the PL images.

## 4. Conclusions

In this work, we investigated the effects of interfacial water on the excitonic behavior of 1L WS_2_ supported on mica. Using mechanical exfoliation, WS_2_ samples could be generated with well-defined mono- and bilayers of interfacial water in addition to essentially water-free areas. PL spectroscopy and wide-field imaging showed that the emission signals from A excitons and their negative trions decreased at distinctively different rates with increasing excitation power. The more pronounced power dependence of the former was attributed to the larger annihilation probability between excitons than trions. Atmosphere-controlled PL imaging also revealed that interfacial water charge-neutralized natively n-doped WS_2_ via the ORR and essentially converted trions into excitons, the latter of which decayed nonradiatively more efficiently than the former. This study will shed light on the role of nanoscopic water in complex low-dimensional materials and eventually contribute to devising their novel functions and related devices.

## Data Availability

Data supporting the reported results can be requested from the corresponding author.

## Supplementary Materials

The following supporting information can be downloaded at: https://www.mdpi.com/article/10.3390/ijms24043492/s1.
