# Supplementary material for: Interfacial-Water-Modulated Photoluminescence of Single-Layer WS2 on Mica
Source: Int J Mol Sci. 2023 Feb 9;24(4):3492. doi: 10.3390/ijms24043492 (PMC9963566; doi:10.3390/ijms24043492)
Supplement: Supplementary file 1 [file ijms-24-03492-s001.zip › ijms-2160830-supplementary.pdf]

# Supporting Information for

# Interfacial-Water-Modulated Photoluminescence of Single-Layer WS2 on Mica

**Yanghee Kim [1,†], Haneul Kang [1,†,] Myeongin Song [1], Hyuksang Kwon [2] and Sunmin Ryu [1,*]**

[1] Department of Chemistry, Pohang University of Science and Technology (POSTECH), Pohang 37673, Republic of Korea
[2] Korea Research Institute of Standards and Science, Daejeon 34113, Republic of Korea
* Correspondence: sunryu@postech.ac.kr

† These authors contributed equally to this work.

## Contents

A. Supporting Data

## A. Supporting Data

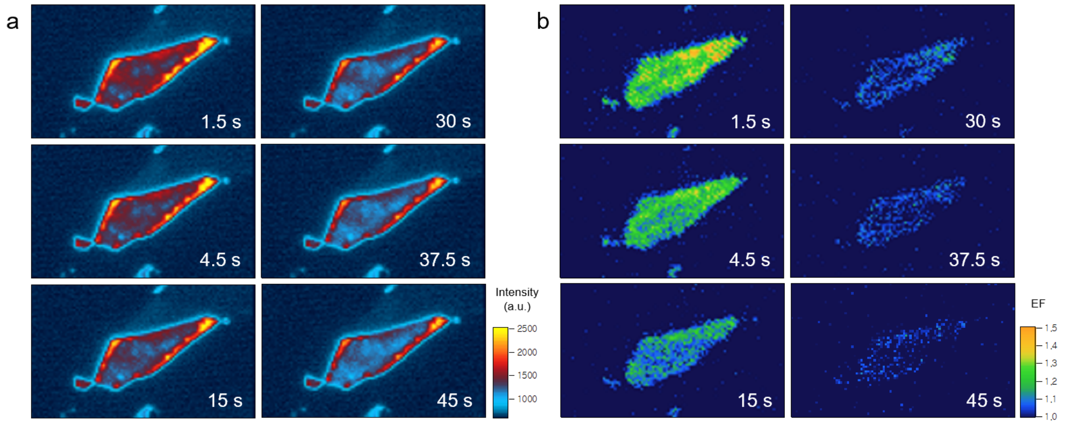


**Figure S1. Undoping of $WS_2$ in Ar gas.** (a & b) Time-lapse PL images (a) and PL enhancement images (b) of the sample in Fig. 4. The sample was pre-equilibrated with $Ar:O_2$ gas sufficiently before exposure to Ar gas at time zero. The enhancement factor (EF) was calculated by normalizing each PL image with respect to that at time zero.
